# Loop-Mediated Isothermal Amplification Assay to Detect Invasive Malaria Vector *Anopheles stephensi* Mosquitoes

**DOI:** 10.3201/eid3009.240444

**Published:** 2024-09

**Authors:** Cristina Rafferty, Gloria Raise, JeNyiah Scaife, Bernard Abongo, Seline Omondi, Sylvia Milanoi, Margaret Muchoki, Brenda Onyango, Eric Ochomo, Sarah Zohdy

**Affiliations:** US President’s Malaria Initiative, Centers for Disease Control and Prevention, Atlanta, Georgia, USA (C. Rafferty, S. Zohdy);; Centers for Disease Control and Prevention, Atlanta (C. Rafferty, G. Raise, J. Scaife, S. Zohdy);; Kenya Medical Research Institute (KEMRI), Kisumu, Kenya (B. Abongo, S. Omondi, S. Milanoi, M. Muchoki, B. Onyango, E. Ochomo)

**Keywords:** malaria, vector-borne infections, Anopheles stephensi, species identification, molecular assay, loop-mediated isothermal amplification, mosquitoes, Kenya, United States

## Abstract

Spread of the *Anopheles stephensi* mosquito, an invasive malaria vector, threatens to put an additional 126 million persons per year in Africa at risk for malaria. To accelerate the early detection and rapid response to this mosquito species, confirming its presence and geographic extent is critical. However, existing molecular species assays require specialized laboratory equipment, interpretation, and sequencing confirmation. We developed and optimized a colorimetric rapid loop-mediated isothermal amplification assay for molecular *An. stephensi* species identification. The assay requires only a heat source and reagents and can be used with or without DNA extraction, resulting in positive color change in 30–35 minutes. We validated the assay against existing PCR techniques and found 100% specificity and analytical sensitivity down to 0.0003 ng of genomic DNA. The assay can successfully amplify single mosquito legs. Initial testing on samples from Marsabit, Kenya, illustrate its potential as an early vector detection and malaria mitigation tool.

In 2012, *Anopheles stephensi*, a primary malaria vector in South Asia, was detected in Djibouti, a country in Africa that was approaching malaria preelimination status (*1*). Unlike typical malaria vectors in Africa, *An. stephensi* mosquitoes can thrive in both urban and rural environments. After the detection in Djibouti, *An. stephensi* mosquitoes were reported in Ethiopia and Sudan in 2016, Somalia in 2019, Nigeria in 2020, Kenya in 2022, and Ghana and Eritrea in 2023 (*2*). The initial detection in Djibouti came during a malaria outbreak (*1*), after which a 36-fold increase in malaria was reported from 2012 to 2020 (*3*). In Dire Dawa, the second largest city in Ethiopia, an unusual dry season outbreak of malaria was reported in 2022, and epidemiologic and entomologic investigations incriminated *An. stephensi* mosquitoes as driving the outbreak (*4*). Furthermore, the species’ insecticide resistance status and unique bionomics present a challenge to proven malaria vector control tools, such as insecticide-treated bed nets and indoor residual spraying (*5*,*6*). Modeling studies have predicted that if *An. stephensi* mosquitoes continue to spread throughout Africa, an additional 126 million persons, predominantly in urban areas, will be at risk for malaria (*7*,*8*). To respond to this threat, the World Health Organization (WHO) launched an initiative to halt the spread of *An. stephensi* mosquitoes (*9*), and global organizations (*10*) and countries have released action plans to encourage enhanced surveillance for the species for early detection in new locations and rapid response to halt spread and mitigate impacts.

Despite efforts to enhance surveillance for *An. stephensi* mosquitoes in Africa, the species was not included in morphologic keys until 2020 (*11*). Therefore, the mosquitoes be missed in routine surveillance activities, and *An. stephensi* mosquitoes could be misidentified as the more common malaria vector *An. gambiae* sensu lato if morphological identification is inadequate (*3*). In addition, reporting a detection of *An. stephensi* mosquitoes in a new country to WHO requires molecular confirmation, which can be challenging in resource-limited settings. Surveillance for *An. stephensi* mosquitoes often requires larval surveys (*12*) because routine malaria vector adult collections are not optimal for the species (*6*) and currently a validated key to identify *An. stephensi* larvae is not available, so larval samples that do not emerge to adults may also require molecular confirmation.

In 2023, a PCR protocol for *An. stephensi* species identification was released and shown to detect *An. stephensi* mosquitoes even among pooled samples, presenting a promising avenue for molecular detection (*13*). However, PCR can be time consuming and limited by molecular laboratory capacity, access to reagents, trained personnel, and assay specificity and interpretation.

Loop-mediated isothermal amplification (LAMP) assays have been used since the 1990s for rapid amplification of gene targets (*14*), resulting in a visual change through fluorescence, turbidity, or color that provides a qualitative indicator of positivity. In this way, LAMP assays function like conventional PCRs, which yield a band (positive) or no band (negative). However, instead of requiring temperature cycling like PCR, LAMP assays produce copies through looped primer sets at 1 consistent temperature, removing the need for a thermal cycler and instead requiring only a heat block, water bath, or any other device that keeps temperature constant. One study even used hand-warmers and a Styrofoam cup to conduct a LAMP assay (*15*). Because the COVID-19 pandemic increased the need for rapid diagnostics, LAMP technology evolved to include colorimetric and dipstick assays (*16*).

To address the challenges that invasive *An. stephensi* mosquito surveillance and corresponding molecular confirmation present, the aim of this study was to develop an easy-to-interpret, rapid colorimetric LAMP-based *Anopheles stephensi* species (CLASS) identification assay, specifically designed and optimized for use in resource-limited settings or for rapid high-throughput screening. To ensure accuracy and feasibility for deployment of the developed assay, we sought to design optimal primers and assay conditions, determine assay sensitivity and pooling strategies, determine assay specificity when compared with congeners or conspecifics, develop direct sample amplification approaches without the need for DNA extraction, compare results between the existing PCR protocol and CLASS assay, and evaluate CLASS on wild-caught, sequence-confirmed invasive *An. stephensi* mosquitoes from Kenya.

## Methods

### LAMP Primer Design and Optimization

**Figure 1:**
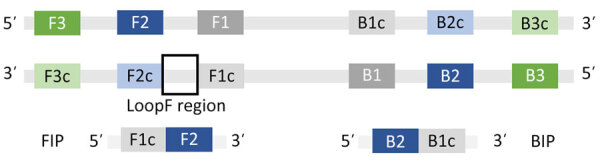
Schematic showing primer design for development of a colorimetric loop-mediated isothermal amplification assay to detect invasive malaria vector *Anopheles stephensi* mosquitoes. Primers were designed by using the NEB LAMP Primer Design Tool version 1.4.1 (New England Biolabs, https://www.neb.com) (*17*). One LAMP primer set contains 5 primers as follows: an outer forward primer (F3), an inner forward primer (FIP), an outer backward primer (B3), an inner backward primer (BIP), and a loop primer (*19*,*20*). Attempts to set fixed primers resulted in no possible loop primer combinations by the program; therefore, we used default parameters and allowed the program to choose primers.

We designed the LAMP primers by using the NEB LAMP Primer Design Tool version 1.4.1 (New England Biolabs, https://www.neb.com) (*17*). We used the internal transcribed spacer 2 rDNA region unique to *An. stephensi* species, using a sequence from GenBank (accession no. MW732931.1) (*18*). One LAMP primer set contains 5 primers as follows: an outer forward primer (F3), an inner forward primer (FIP), an outer backward primer (B3), an inner backward primer (BIP), and a loop primer (Figure 1) (*19*,*20*). Attempts to set fixed primers resulted in no possible loop primer combinations by the program; therefore, we used default parameters and allowed the program to choose primers. 

Of 4 possible primer sets, 2 contained primers in the species-specific region. We tested those primers across a temperature gradient using 2 sets of differing concentrations. Initial test concentrations were adapted from NEB kit manufacturer recommendations, and primer concentrations were based on an *An. gambiae* species identification LAMP assay (*21*). One primer set showed positive, consistent results and minimum cross-reactivity to other species. We then further optimized that primer set for maximum specificity (Table 1; Figure 1).

**Table 1:** Looped primers designed by using the NEB LAMP Primer Design Tool targeting *Anopheles stephensi* mosquito ITS2 sequence regions for development of colorimetric LAMP assay to detect invasive malaria vector *An. stephensi* mosquitoes*

Sequence	5′	3′	Primer sequence	Primer concentration
F3	333	351	ATTGCACGGGGACTTCCA	5 µM
B3	504	524	GCCTACAGACTCCACTGTCA	5 µM
FIP			CGACTGCAACTGTATGCGAGGACGGGTCGAGTAACACTTGC	20 µM
BIP			CCGTGTGGGTGAGTGAGGTTAGAATGATGCGACGGGAGAAG	20 µM
LF	383	401	AAGATACGAGCGCGTTGGG	10 µM
F1c†	402	423	CGACTGCAACTGTATGCG	
F2†	359	380	GGACGGGTCGAGTAACACTTGC	
B2†	439	461	CCGTGTGGGTGAGTGAGGTTAG	
B1c†	485	502	AATGATGCGACGGGAGAAG	

*ITS, internal transcribed spacer; LAMP, loop-mediated isothermal amplification; NEB, New England Biolabs, https://www.neb.com. †Primers not needed for LAMP assay.

### Insectary-Reared *An. stephensi* and Other Mosquito Species

We obtained larvae and adult insectary-reared and maintained colony mosquitoes from 8 distinct non–*An. stephensi Anopheles* species, 3 strains of *An. stephensi* mosquitoes of different origins (STE2 from India, SDA 500 from Pakistan, UCI from India), and one *Aedes* (*Ae. aegypti*) mosquito. Mosquitoes came from the Malaria Research and Reference Reagent Resource Center through BEI Resources (Table 2) (*22*).

**Table 2:** Mosquito species and corresponding strains and catalog numbers used in development of colorimetric loop-mediated isothermal amplification assay to detect invasive malaria vector *Anopheles stephensi* mosquitoes*

Species	MRA BEI reference no.	Strain name
*Anopheles gambiae* sensu stricto	112	G3
*An. gambiae* s.s./*An. coluzzii* hybrid	334	RSP
*An. coluzzii*	1280	AKDR
*An. arabiensis*	856	DONGOLA
*An. stephensi*	1323	UCI
*An. stephensi*	NA	SDA500
*An. funestus* s.s	NA	FANG
*An. quadriannulatus*	1155	SANGWE
*An. dirus*	700	WRAIR
*An. merus*	1156	MAF
*An. stephensi*	128	STE2
*Aedes aegypti*	734	ROCK

*BEI, BEI Resources, https://www.beiresources.org; MRA, Malaria Repository Acquisition; NA, not applicable.

### DNA Extraction

We extracted DNA from whole adult, single mosquito leg, and whole third instar larva by using the Extracta DNA Prep for PCR Kit (Quantabio Beverly, https://www.quantabio.com), adapted as follows: mosquito material added to a tube containing either 25 µL (for a single leg) or 50 µL (for a whole adult or larva) of Quantabio Extraction Reagent and incubated at 95°C for 30 minutes. We added an equal volume of Quantabio Stabilization Buffer and stored DNA at −20°C until further analysis. For the pooled species sample, we combined 1 µL of DNA from 9 DNA extractions of non–*An. stephensi* mosquitoes and 1 µL of DNA extracted from *An. stephensi* mosquitoes (STE2) in a microfuge tube and mixed contents.

### CLASS Assay

We carried out CLASS reactions by using the NEB WarmStart Colorimetric LAMP 2X Master Mix (New England Biolabs), according to manufacturer recommendations but optimized as follows: 1 µL of genomic DNA was added to 12.5 µL of WarmStart Colorimetric LAMP 2X Master Mix and 10X primers at final concentrations of 5 µM of B3 and F3 primers, 20 µM of BIP and FIP primers, and 10 µM of LF primer. We added molecular-grade water to reach a final volume of 25 µL. We placed reaction tubes in a thermal cycler at 65°C for 30 minutes and inspected visually for color change, where positive amplification appears yellow and negative remains pink. We tested primers on extracted DNA from 12 assorted insectary-reared adults and larvae, including 3 *An. stephensi* strains (STE2, SDA500, and UCI), 14 field-collected specimens including 3 sequence-confirmed *An. stephensi* mosquitoes, and DNA from pooled species. We included a no-DNA control in each run of the assay.

### Analytical Sensitivity 

To test analytical sensitivity, we made a serial dilution (1:10) of DNA extract from a whole UCI *An. stephensi* mosquito and determined starting DNA concentration by using a NanoDrop 2000c spectrophotometer (Thermo Scientific, https://www.thermofisher.com) on 1 μL of DNA extract. For each concentration, no color change (pink) indicated a negative result and color change (yellow) a positive result. We ran samples from the dilution series in triplicate with no full change detected and 2 additional dilutions to determine the sensitivity cutoff.

### Specificity Determination

We tested optimized primers against 12 laboratory anopheline strains that included 3 *An. stephensi* strains (SDA500, STE2, UCI) and 1 *Ae. aegypti* strain (Table 2). We subsequently tested the assay against 96 individual mosquitoes from each *An. stephensi* laboratory strain and 48 *An. gambiae*, *An. coluzzii*, *An. arabiensis*, *An. funestus*, and *Ae. aegypti* laboratory-reared samples for specificity and cross reactivity. We ran all reactions in triplicate to generate data on cross-reactivity with other species and specificity to *An. stephensi.* We included 3 *An. stephensi* strains to determine variations in target specificity across *An. stephensi* mosquitoes of different origins.

### Samples for LAMP Amplification

To determine whether DNA extract is needed to run the CLASS assay or if tissue (mosquito leg, full larva, full adult mosquito) or pooled DNA amplify, we inserted single legs from insectary-reared mosquitoes directly into the master mix and compared the results with DNA extracted from a single leg. Because *An. stephensi* samples are often collected as larvae, we also tested the assay by immersing a whole larva into the master mix and using extracted DNA from a single larva. We also partially tested eDNA by using 1 µL using larval pan water in lieu of extracted DNA. We further tested the assay against whole adult mosquitoes and compared results with whole adult mosquito DNA. In addition, we tested pooled DNA extract from whole adult mosquitoes and from individual legs from 9 mosquito strains and 1 *An. stephensi* strain.

### Conventional PCR Comparison

We compared CLASS results with those from a conventional *An. stephensi* species identification PCR assay by using previously described methods (*13*). We adapted the method as follows: 2X Quantabio Accustart PCR mix, 10 µM of each primer, molecular water to reach a final volume of 20 µL, and 1 µL of the extracted DNA from same species used in the CLASS assay.

### CLASS Assay Validation on *An. stephensi* Mosquitoes from Kenya

We ran sequence-confirmed DNA extracted from wild-caught *An. stephensi* mosquitoes from Kenya (GenBank accession nos. OQ275144–6 and OQ878216–8) using the CLASS assay (*23*). We additionally tested 55 wild-caught samples collected from Marsabit, Kenya, in 2023 that previously failed to amplify via conventional PCR (*24*). DNA extracted at the Kenya laboratory was dried and shipped to the US Centers for Disease Control and Prevention (Atlanta, Georgia, USA), where samples were resuspended in 25 µL of PCR-grade water and stored at −20°C until processed.

## Results

### LAMP Primer Design and Assay Optimization

We tested the 4 primer sets suggested by the NEB LAMP Primer Design Tool version 1.4.1 against extracted DNA from 3 *An. stephensi* insectary strains and 8 other *Anopheles* species: *An. gambiae* s.s., *An. coluzzii*, *An. arabiensis*, *An. gambiae/coluzzii* hybrid, *An. funestus*, *An. quadriannulatus*, *An. dirus*, and *An. merus* (*17*). We tested the 2 primer sets (P2L-45 and P26L2) that showed color change for *An. stephensi* samples and minimum cross-reactivity among other species with varying concentrations at 3 incubation times: 15, 30, and 45 minutes. No color change was detected at 15 minutes, but specificity was affected at 45 minutes, confirming 30 minutes as the ideal assay incubation time. We analyzed 384 reactions in duplicate (768 total reactions) using 8 different primer concentration combinations, and we chose primer set P26L2 for its consistent sensitivity and specificity (Table 3). We tested the chosen primers and respective concentration combinations using a temperature gradient (57°C, 61°C, 65°C, 69°C, 73°C, and 83°C) through 176 separate reactions. Results confirmed that primer concentration combination B3 at 65°C yielded the most consistent and specific results (Table 3). Amplification occurred at 69°C and 73°C, but specificity was inconsistent. We observed no amplification at higher or lower temperatures.

**Table 3:** Primer set candidates tested with different concentrations to determine effects on sensitivity and specificity of colorimetric loop-mediated isothermal amplification assay to detect invasive malaria vector *Anopheles stephensi* mosquitoes

Concentration combination							Primer set*				
	Primer concentration						P26L2			P2L-45	
	F3	B3	FIP	B IP	Loop primer		Sensitivity	Specificity		Sensitivity	Specificity
A1	2 µM	2 µM	16 µM	16 µM	4 µM		X	✓		X	X
A2	4 µM	4 µM	16 µM	16 µM	4 µM		✓	X		X	X
A3	2 µM	2 µM	32 µM	32 µM	4 µM		✓	X		X	X
A4	2 µM	2 µM	16 µM	16 µM	8 µM		X	✓		X	X
B1	5 µM	5 µM	40 µM	40 µM	10 µM		✓	X		✓	X
B2	2.5 µM	2.5 µM	40 µM	40 µM	10 µM		✓	X		X	✓
B3	5 µM	5 µM	20 µM	20 µM	10 µM		✓	✓		X	X
B4	5 µM	5 µM	40 µM	40 µM	5 µM		✓	X		X	X

*For each primer set, a check mark indicates consistent results and an X inconsistent results.

Repeated time-interval testing of 384 samples with the chosen primers showed no amplification before 25 minutes, optimum amplification at 30 minutes, and a decrease of specificity after 35 minutes. Consequently, we adopted a 30-minute incubation period for the assay. Once incubation stopped (by removal from the heat source), the product and color change remained stable and unaltered at room temperature for >12 weeks.

### CLASS Assay Analytical Sensitivity

To test the sensitivity of the CLASS assay, we performed serial dilutions (1:10) of initial DNA extract to a concentration of 311.6 ng, which resulted in 100% positive color change to yellow. Positive color change was repeatedly observed at >0.0003 ng; concentrations <0.0003 ng yielded positive color change, but changes occurred inconsistently (33.3% of the time). Because lower concentrations did not yield 100% color change, the assay sensitivity threshold established in this study is as low as 0.0003 ng, 1,000 times lower than what is found in typical DNA extract from a single leg (Table 4).

**Table 4:** Assessment of assay sensitivity for development of colorimetric loop-mediated isothermal amplification assay to detect invasive malaria vector *Anopheles stephensi* mosquitoes*

Template dilution	Template concentration, ng	UCI
NTC	NA	3/3 negative
1	311.6	3/3 positive
10	31.16	3/3 positive
1 × 10^2^	3.12	3/3 positive
1 × 10^3^	0.312	3/3 positive
1 × 10^4^	0.031	3/3 positive
1 × 10^5^	0.003	3/3 positive
1 × 10^6^	0.0003	3/3 positive
1 × 10^7^	0.00003	1/3 positive, 2/3 negative

*Assessment was done using a serial (1:10) dilution from DNA extract containing 311.6 ng of *Anopheles stephensi* (UCI) whole mosquito DNA and resulted in 100% positive color change down to 0.0003 ng. Each dilution test was performed in triplicate. Color change at 1 × 10^7^ was inconsistent, and samples with DNA concentrations <0.0003 were less likely to result in a positive color change and did not display as clear pink or yellow. NA, not applicable; NTC, no template control; UCI, *An. stephensi* laboratory colony (BEI Resources, https://www.beiresources.org).

### CLASS Assay Specificity and Cross-Reactivity

**Figure 2:**
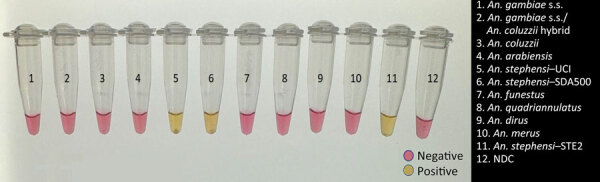
Visualization of testing using a colorimetric loop-mediated isothermal amplification assay to detect invasive malaria vector *Anopheles stephensi* mosquitoes. Positive samples show a color change to yellow, whereas negative samples and control remain pink. Samples were visualized on a white background and photographed on a standard light box. NDC, no DNA template control; UCI, *An. stephensi* laboratory colony (BEI Resources, https://www.beiresources.org).

We tested the optimized P26L2 primers (Table 1) against extracted DNA from 11 insectary strains, including 3 *An. stephensi* mosquitoes*.* We ran the assay 11 separate times with different extracted DNA from single whole-colony mosquitoes for a total of 132 reactions (Figure 2). We further assessed specificity by testing DNA from 96 individual mosquitoes from each *An. stephensi* laboratory strain; 100% of the samples yielded a positive result. We determined cross-reactivity by sampling DNA from 48 *An. gambiae*, 48 *An. coluzzii*, 48 *An. arabiensis*, 48 *An. funestus*, and 48 *Ae. aegypti* whole mosquitoes and analyzing. None (0%) of the non–*An. stephensi* strains showed color change. We ran all specificity assays in triplicate (Table 5).

**Table 5:** Specificity testing for colorimetric loop-mediated isothermal amplification assay to detect invasive malaria vector *Anopheles stephensi* mosquitoes*

Species	Colony name	No. mosquitoes	Experiment 1	Experiment 2	Experiment 3
*An. stephensi*	STE2	96	Positive (96/96)	Positive (96/96)	Positive (96/96)
*An. stephensi*	SDA500	96	Positive (96/96)	Positive (96/96)	Positive (96/96)
*An. stephensi*	UCI	96	Positive (96/96)	Positive (96/96)	Positive (96/96)
*An. gambiae* senso stricto	G3	48	Negative (0/48)	Negative (0/48)	Negative (0/48)
*An. coluzzii*	AKDR	48	Negative (0/48)	Negative (0/48)	Negative (0/48)
*An. arabiensis*	DONGOLA	48	Negative (0/48)	Negative (0/48)	Negative (0/48)
*An. funestus* s.s	FANG	48	Negative (0/48)	Negative (0/48)	Negative (0/48)
*Aedes aegypti*	ROCK	48	Negative (0/48)	Negative (0/48)	Negative (0/48)
Total no. samples		528			
Total no. reactions			1,584		

*To determine specificity, we ran the essay in triplicate on extracted DNA from 96 individual mosquitoes from each of the 3 *An. stephensi* insectary strains and DNA extracted from 48 individual mosquitoes from each species of *An*. *gambiae* sensu stricto, *An. coluzzii*, *An. arabiensis, An. funestus,* and *Ae. aegypti.*

### CLASS Assay Testing of Mosquito Tissue, DNA Extract, and DNA Pooling

**Figure 3:**
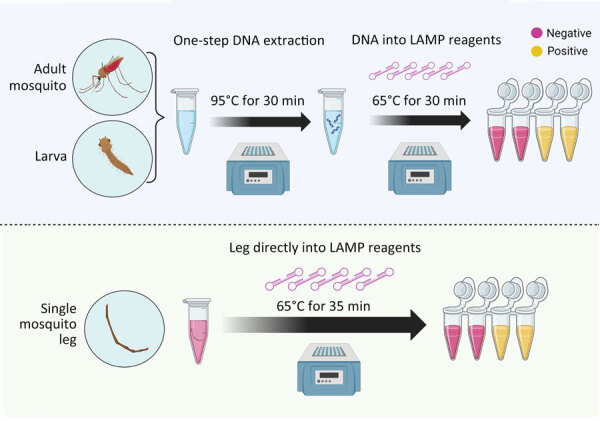
Schematic for colorimetric loop-mediated isothermal amplification assay to detect invasive malaria vector *Anopheles stephensi* mosquitoes. Top: DNA from any mosquito source directly placed in the colorimetric master mix are incubated at 65°C for 30 minutes to obtain a yellow color change, showing a positive sample. The assay shows high sensitivity and specificity when DNA extract from adult or larval mosquitoes is used. Bottom: Assay can also be used directly on a single mosquito leg, without the need for DNA extraction, by adding a 5-minute extension to incubation time (i.e., 35 minutes). Schematic produced using Biorender (https://www.biorender.com).

Using DNA extract from a single leg resulted in color change in *An. stephensi* mosquitoes, with no cross-reactivity with other tested species. Inserting a single mosquito leg straight into the master mix also successfully amplified after optimization, but at a 35-minute incubation time (Figure 3). When testing a whole larva or whole adult mosquito, the assay had low specificity, and yielded cross-reactivity; however, the use of DNA extract from a single larva or mosquito from 11 *Anopheles* colony strains, including 3 *An. stephensi* strains and 1 *Ae. aegypti* strain, resulted in species-appropriate color change (Figure 3). Limited testing on larval pan water yielded inconclusive results. Although the CLASS assay was able to identify *An. stephensi* from larval pan water and not from other anopheline larval water, results showed cross-reactivity with *Ae. aegypti*.

### CLASS Assay Specificity in Field Samples and Comparison with Conventional PCR

**Figure 4:**
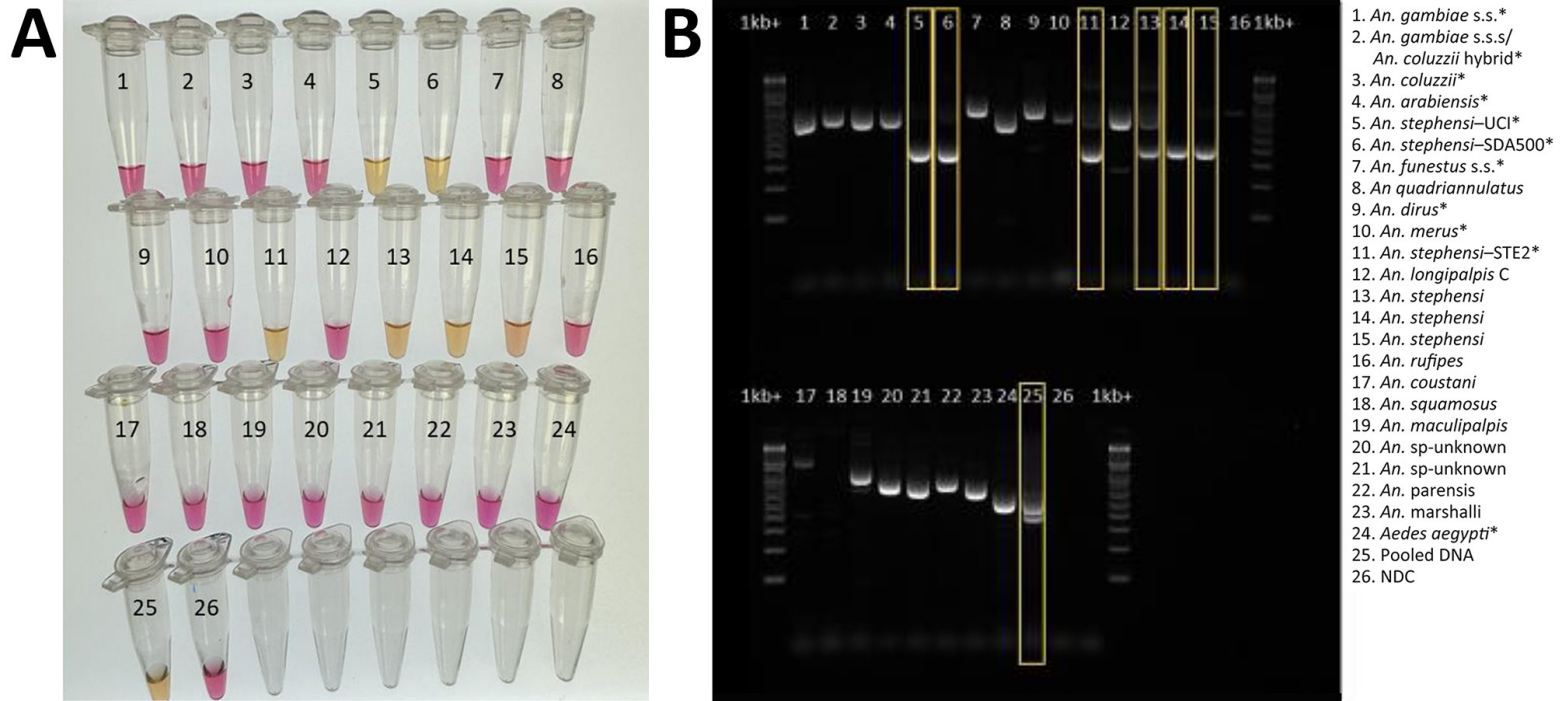
Validation of colorimetric loop-mediated isothermal amplification assay to detect invasive malaria vector *Anopheles stephensi* mosquitoes. A) Results using new assay. B) Results using existing *An. stephensi* PCR (*13*); yellow boxes indicate *An. stephensi* products. Conventional PCR resulted in difficult-to-interpret gel bands for *An. longipalpis* C, *Aedes aegypti*, and *An. coustani* samples, similar to *An. stephensi* samples, and inconsistently produced double bands (positive detection) on sequence-confirmed *An. stephensi* samples from Kenya. Asterisks (*) in key indicate samples from insectary-reared mosquitoes. Samples 12–23 came from sequence-confirmed field-collected specimens. Sample 25 contained a pool of assorted mosquito DNA species, in which *An. stephensi* was represented 1:10. For both assays, 1 µL extracted DNA was used. NDC, no DNA template control; UCI, *An. stephensi* laboratory colony (BEI Resources, https://www.beiresources.org).

Sequence-confirmed *An. stephensi* samples from Kenya positively amplified using CLASS, and no cross-reactivity was seen with other *Anopheles* species. *An. stephensi* sampled in pooled DNA from 9 colony-reared species (*An. gambiae* s.s., *An. coluzzii*, *An. gambiae/coluzzii* hybrid, *An. arabiensis*, *An. funestus*, *An. quadriannulatus*, *An. merus*, *An. dirus*, and *Ae. aegypti*) and 1 *An. stephensi* sample (SDA 500) also amplified using CLASS. Conventional PCR resulted in difficult-to-interpret gel bands for *An. longipalpis* C, *Ae. aegypti*, and *An. coustani* samples, similar to *An. stephensi* samples, and inconsistently produced double bands (positive detection) on sequence-confirmed *An. stephensi* samples from Kenya (Figure 4, panel B).

### CLASS Assay Testing of Field Samples from Marsabit, Kenya

CLASS assay testing of 55 wild-caught *Anopheles* samples from Marasabit, Kenya, successfully identified the 9 cytochrome c oxidase subunit I sequence-confirmed *An. stephensi* samples. Furthermore, no cross-reactivity was observed with the other species or unknown samples (Table 6). Twelve nonamplified samples in the sample set during barcoding also tested negative by the CLASS assay.

**Table 6:** Testing of wild *Anopheles* spp. mosquitoes collected in Marsabit, Kenya, in 2022 and 2023 that failed to amplify during routine species assays as part of development of colorimetric LAMP assay to detect invasive malaria vector *Anopheles stephensi* mosquitoes*

Site	Collection method	Date of collection	Sequence-confirmed species	Colorimetric LAMP assay result
Marsabit	Light trap	2023 Feb	*An. gambiae* senus lato	Negative (0/2)
Marsabit	Light trap	2023 Feb	*An. gambiae* s.l.	Negative (0/2)
Marsabit	Larvae	2023 Feb	*An. dthali*	Negative (0/2)
Marsabit	Larvae	2023 Feb	*An. gambiae* s.l.	Negative (0/2)
Marsabit	Larvae	2023 Feb	*An. dthali*	Negative (0/2)
Marsabit	Larvae	2023 Feb	*Culex peresiguus*	Negative (0/2)
Marsabit	Larvae	2023 Feb	*An. dthali*	Negative (0/2)
Marsabit	Adults	2022 Dec	NA	Negative (0/2)
Marsabit	Adults	2022 Dec	*An. gambiae* s.l.	Negative (0/2)
Marsabit	Adults	2022 Dec	*An. stephensi*	Positive (2/2)
Marsabit	Adults	2022 Dec	NA	Negative (0/2)
Marsabit	Adults	2022 Dec	*An. gambiae* s.l.	Negative (0/2)
Marsabit	Adults	2022 Dec	*An. dthali*	Negative (0/2)
Marsabit	Adults	2022 Dec	*An. gambiae* s.l.	Negative (0/2)
Marsabit	Adults	2022 Dec	NA	Negative (0/2)
Marsabit	Adults	2022 Dec	NA	Negative (0/2)
Marsabit	Adults	2022 Dec	NA	Negative (0/2)
Marsabit	Adults	2022 Dec	*An. dthali*	Negative (0/2)
Marsabit	Adults	2022 Dec	*An. stephensi*	Positive (2/2)
Marsabit	Adults	2022 Dec	NA	Negative (0/2)
Marsabit	Adults	2022 Dec	NA	Negative (0/2)
Marsabit	Adults	2022 Dec	*An. gambiae* s.l.	Negative (0/2)
Marsabit	Adults	2022 Dec	Other	Negative (0/2)
Marsabit	Adults	2022 Dec	NA	Negative (0/2)
Marsabit	Larvae	2023 May	*An. gambiae* s.l.	Negative (0/2)
Marsabit	Larvae	2023 May	*An. pretoriensis*	Negative (0/2)
Marsabit	Larvae	2023 May	*An. stephensi*	Positive (2/2)
Marsabit	Larvae	2023 May	*An. gambiae* s.l.	Negative (0/2)
Marsabit	Larvae	2023 May	*An. pretoriensis*	Negative (0/2)
Marsabit	Larvae	2023 May	NA	Negative (0/2)
Marsabit	Larvae	2023 May	*An. gambiae* s.l.	Negative (0/2)
Marsabit	Larvae	2023 May	*An. gambiae* s.l.	Negative (0/2)
Marsabit	Larvae	2023 May	*An. stephensi*	Positive (2/2)
Marsabit	Larvae	2023 May	*An. stephensi*	Positive (2/2)
Marsabit	Larvae	2023 May	Other	Negative (0/2)
Marsabit	Larvae	2023 May	*An. gambiae* s.l.	Negative (0/2)
Marsabit	Larvae	2023 May	*An. stephensi*	Positive (2/2)
Marsabit	Larvae	2023 May	*An. gambiae* s.l.	Negative (0/2)
Marsabit	Larvae	2023 May	*An. stephensi*	Positive (2/2)
Marsabit	Larvae	2023 May	NA	Negative (0/2)
Marsabit	Larvae	2023 May	*An. pretoriensis*	Negative (0/2)
Marsabit	Larvae	2023 May	*An. pretoriensis*	Negative (0/2)
Marsabit	Larvae	2023 May	*An. pretoriensis*	Negative (0/2)
Marsabit	Larvae	2023 May	NA	Negative (0/2)
Marsabit	Larvae	2023 May	*An. stephensi*	Positive (2/2)
Marsabit	Larvae	2023 May	*An. gambiae* s.l.	Negative (0/2)
Marsabit	Larvae	2023 May	*An. stephensi*	Positive (2/2)
Marsabit	Larvae	2023 May	*An. gambiae* s.l.	Negative (0/2)
Marsabit	Larvae	2023 May	*An. pretoriensis*	Negative (0/2)
Marsabit	Larvae	2023 May	Other	Negative (0/2)
Marsabit	Larvae	2023 May	*An. gambiae* s.l.	Negative (0/2)
Marsabit	Larvae	2023 May	NA	Negative (0/2)
Marsabit	Larvae	2023 May	*An. gambiae* s.l.	Negative (0/2)
Marsabit	Larvae	2023 May	*An. pretoriensis*	Negative (0/2)
Marsabit	Larvae	2023 May	*An. pretoriensis*	Negative (0/2)

*Samples were sequenced using cytochrome c oxidase subunit I sequence testing to confirm species identification, and then DNA extract from the samples was run in duplicate through the colorimetric LAMP assay. Results showed 100% concurrence between the LAMP assay results and Sanger sequencing in determining which specimens were *An. stephensi* and which were not. LAMP, loop-mediated isothermal amplification; NA, no amplification occurred; other, other (non-*Anopheles*) mosquito species.

## Discussion

Molecular species identification of malaria vectors is pivotal for effective control and elimination strategies, particularly because malaria-vector mosquitoes often cannot be morphologically identified to the species level. Of increasing complexity is the introduction of invasive species, such as *An. stephensi*, that are not included in traditional identification keys (*25*) and thus can be easily misidentified. In addition, to confirm the presence of *An. stephensi* mosquitoes on the WHO *An. stephensi* Threats Map (*26*), molecular confirmation through Sanger sequencing (*9*) is required.

We developed a rapid 1-step colorimetric LAMP assay for species identification of *An. stephensi* mosquitoes to accelerate tracking this species across Africa or in locations where it is endemic. The CLASS identification assay provides a precise and reliable means of *An. stephensi* identification. Our findings indicate high sensitivity and specificity of the assay, whether *An. stephensi* samples were mixed in a pool of 10 other species or validated against 8 species, including 3 unique insectary-reared strains and individual wild-caught invasive *An. stephensi* samples. No false positives or false negatives were observed. When we conducted a dilution series to determine analytical sensitivity, even at 0.0003 ng of DNA, the CLASS assay detected *An. stephensi* DNA. Thus far, the specificity remains 100% when other species are processed through the assay. The ability to differentiate between various *Anopheles* species, especially those with differing vectorial capacities or behaviors, is indispensable for tailoring interventions to specific vector populations (*27*).

The CLASS assay can be run using a single mosquito leg or DNA extract from an adult or larval mosquito (Figure 3). DNA extracted from a leg is preferable so specimens can remain largely intact for further curation, sequencing, and storage. The use of an entire mosquito or larva is highly discouraged because it yields nonspecific results and prevents further follow-up and species confirmation. For WHO submission and confirmation, sequencing-positive specimens are still encouraged; however, the CLASS assay provides a rapid, high-throughput, field-friendly screening tool for an initial detection of *An. stephensi* mosquitoes. Although initial testing of larval pan water yielded inconclusive results, possibly because of *Aedes* excessive larval shedding in the water, findings suggest the potential for additional exploration using CLASS to examine environmental DNA or large pools of specimens to yield further information about potential cross-reactivity with other species in natural settings.

A conventional PCR to support molecular detection of *An. stephensi* mosquitoes exists (*13*); however, in settings where facilities and trained personnel are limited, conventional PCR can be challenging. That PCR also has multiple primers, and thus, interpreting results can be a challenge if one or both bands are absent. In our study, insectary and field *Anopheles* samples run through the conventional PCR showed gel bands that could be misinterpreted as false-positive or -negative. Even insectary-reared *An. stephensi* samples produced inconclusive results using that assay (Figure 4, panel B). External laboratories have also reported nonamplification using the assay on samples later confirmed to be *An. stephensi* through sequencing (*23*). Follow-up PCR and Sanger sequencing validation are still critical, but our findings support the need for a robust *An. stephensi* assay that is simple to interpret.

The CLASS assay showed promising results when field-caught samples from Kenya were tested. Although testing on those samples used DNA, this assay’s ability to test single legs without extraction and using simple equipment suggests potential for screening large numbers of wild mosquitoes in remote settings. With additional field deployment and validation, data may be generated to potentially consider the CLASS assay as a species confirmation tool if confirmed sensitivity and specificity continue to fall within an 85% CI. In addition, alternate LAMP detection chemistries using the primers we describe could be adapted to ensure assay capacity in all settings without relying on a single company and master mix and without concern for reagent quality affecting pH change.

LAMP assay technology improved because of the need for rapid cost-effective diagnostics during the COVID-19 pandemic. Because phenol-based colorimetric LAMP assays are now widely adaptable, opportunities exist beyond *An. stephensi* species identification, such as for *An. arabiensis*, a common malaria vector in Africa currently requiring PCR for species confirmation (*28*). In some locations, *An. arabiensis* mosquitoes are the primary malaria vector, and a colorimetric LAMP screening tool could be used to rapidly distinguish non–*An. arabiensis* samples for further molecular confirmation. The first detection of *An. stephensi* mosquitoes in Ethiopia occurred when *An. gambiae* sensu lato mosquitoes did not amplify as *An. arabiensis* mosquitoes and sequencing revealed invasive *An. stephensi* mosquitoes instead (*29*).

Molecular species identification provides crucial data for epidemiologic surveillance. Real-time data on vector distribution and density guide the implementation of vector control methods, such as insecticide-treated bed nets and indoor residual spraying, ensuring that resources are used effectively to curb malaria transmission (*30*). Early detection, assisted by rapid assays like the CLASS assay, is critical for initiating timely responses to invasive vector populations (*7*).

The significance of accurate molecular identification of vector species extends beyond invasive *An. stephensi*. Long-term research and malaria program initiatives, guided by species identification data, enable program managers and scientists to study vector biology, behavior, and genetics. Such insights are invaluable for developing effective control tools and strategies. In addition, policy formulation relies heavily on accurate surveillance data. Molecular surveillance of vectors like *An. stephensi* informs policy decisions at regional, national, and international levels, ensuring a coordinated and effective response to malaria (*31*). Accurate molecular identification not only aids in understanding the geographic distribution of vectors but also assists in predicting potential disease outbreaks, enabling public health authorities to proactively allocate resources and plan interventions (*32*).

In conclusion, molecular species identification of malaria vectors, particularly in the context of invasive species such as *An. stephensi*, is indispensable to ensure gains made in global malaria control and elimination over the last few decades are not lost. Developing rapid, cost-effective assays, such as the CLASS assay, marks a substantial advancement in the ability to detect *An. stephensi* mosquitoes early in new locations, enabling rapid vector control response. This assay has potential as a screening tool to monitor the spread of the vector species. This tool is field adaptable and can be used in resource-limited settings so that laboratory capacity is not a bottleneck preventing countries from detecting and reporting the presence of *An. stephensi* mosquitoes. By combining accurate molecular identification of *An. stephensi* mosquitoes with adaptive interventions, policymakers, researchers, and public health officials can work collaboratively to mitigate the effect of this invasive malaria vector and continue to work toward a malaria-free future.

Appendix Additional information for rapid, cost-effective, colorimetric LAMP assay to detect invasive malaria vector *Anopheles stephensi* mosquitoes.
